# Readmission Trends and Outcomes of Transcatheter Edge-to-Edge Repair of Mitral Regurgitation With and Without Anemia

**DOI:** 10.7759/cureus.59101

**Published:** 2024-04-26

**Authors:** Bandar Al Yami, Yasar Sattar, Waleed Alruwaili, Nouraldeen Manasrah, Varun Victor, Jawad Basit, Mustafa Bdiwi, Anoop Titus, Neel N Patel, Anas A Alharbi, David Song, Sameer Raina, M. Chadi Alraies

**Affiliations:** 1 Internal Medicine, West Virginia University School of Medicine, Morgantown, USA; 2 Internal Medicine, Icahn School of Medicine at Mount Sinai, New York City, USA; 3 Cardiology, West Virginia University Heart and Vascular Institute, Morgantown, USA; 4 Internal Medicine, Medical College of Georgia, Augusta University, Augusta, USA; 5 Internal Medicine, Canton Medical Education Foundation, Canton, USA; 6 Department of Medicine, Rawalpindi Medical University, Rawalpindi, PAK; 7 Department of Cardiology, Holy Family Hospital, Rawalpindi, PAK; 8 Internal Medicine, Wayne State University Detroit Medical Center, Detroit, USA; 9 Internal Medicine, Saint Vincent Hospital, Worcester, USA; 10 Internal Medicine, New York Medical College/Landmark Medical Center, Valhalla, USA; 11 Medicine, B. J. Medical College, Ahmedabad, IND; 12 Medicine, West Virginia University, Morgantown, USA; 13 Internal Medicine, Icahn School of Medicine at Mount Sinai, Elmhurst Hospital Center, New York, USA; 14 Cardiology, Wayne State University Detroit Medical Center, Detroit, USA

**Keywords:** healthcare outcomes, mitral regurgitation (mr), readmission rate, anemia, transcatheter edge-to-edge repair

## Abstract

Background: Anemia is associated with worse clinical outcomes in cardiac patients. We aim to investigate the clinical outcomes and readmission rates in anemic patients undergoing transcatheter edge-to-edge repair (TEER) for severe mitral valve regurgitation (MR).

Methods: The National Readmissions Database (NRD) from 2015 to 2018 was queried using the ICD-10 codes to identify patients admitted for TEER. Patients were divided into anemic and non-anemic sub-groups. Univariate and multivariate analyses were performed. Cardiovascular outcomes were assessed between cohorts at index admission and readmissions at 30, 90, and 180 days. STATA v.17 was used for analysis (StataCorp LLC, Texas, USA).

Results: Our final cohort included 28,995 patients who had undergone TEER in the United States between 2016 and 2019. About 1,434 (4.9%) had a diagnosis of anemia. The mean age of patients who had TEER with anemia and TEER without anemia was 76.9 ± 10.8 vs. 77.7 ± 10.2, respectively. In the adjusted model, anemic patients had higher odds of acute kidney injury (AKI) (aOR 2.21; 95% [CI 1.81-2.6; p<0.001]), HF (aOR 1.75; 95% [CI 1.28-2.3; p<0.001]), myocardial infarction (MI) (aOR 1.54; 95% [CI 1.01-2.33; p<0.041]), major adverse cardiac and cerebrovascular events (MACCE) (aOR 1.72; 95% [CI 1.2-9-2.3; p<0.001]), and net adverse event (aOR 1.85; 95% [CI 1.32-2.59; p<0.001]). The anemic group's readmission rate was overall higher at 30, 90, and 180 days from 2016 to 2019.

Conclusion: Anemia was associated with increased adverse clinical outcomes and more extended hospital stays in patients with anemia who had undergone TEER procedures compared to the non-anemic group.

## Introduction

Mitral valve regurgitation (MR) is the most common valvular disorder in the United States, affecting more than 2 million individuals, with an estimated prevalence of 10% in patients over 75 [1]. It can be primarily due to an abnormality that affects the mitral valve apparatus or secondary to left ventricular dysfunction [2]. The transcatheter edge-to-edge repair (TEER) procedure was associated with low morbidity and mortality rates and acute MR reduction to <2+ grade in most patients in the initial EVEREST (endovascular valve edge-to-edge repair study) study [3]. According to the EVEREST II trial, the same procedure was associated with superior safety compared to conventional surgery and similar improvements in clinical outcomes [4]. It spares patients' long-term adverse consequences of valve replacement and improves left ventricular function. In addition, it is associated with an improvement in MR severity and survival compared to medical therapy in high-risk groups [5,6]. Despite this, there is a degree of variability in positive outcomes, and some patients demonstrate only mild improvement [7]. This is likely because patients undergoing mitral clipping are an older population with various comorbidities. 

Anemia is a common comorbidity in patients with cardiac diseases that has been associated with adverse clinical outcomes. Patients with anemia undergoing cardiac surgery had a higher morbidity and mortality rate [7]. For example, patients with anemia who had transcatheter aortic valve replacement (TAVR) had a higher one-year mortality rate compared to patients without anemia [8]. Furthermore, heart failure patients with anemia had a worse quality of life with lower physical activity tolerance [9]. Given that most patients undergoing percutaneous mitral valve repair (PMVR) have severe heart failure, we attribute this to the fact that anemia is prominent in these patients and negatively affects clinical outcomes. Anemia was an independent predictor of moderate or severe functional MR in non-ischemic dilated cardiomyopathy (DCM) patients [10].

Intravascular hemolysis is associated with subclinical anemia in patients with primary mitral regurgitation [11]. Pre-TEER procedural anemia was found to be strongly related to mortality rates and poor outcomes during mid-term follow-up [12]. With this background, we aimed to investigate the clinical effects of anemia in patients undergoing TEER for severe MR.

## Materials and methods

Study design

This was a retrospective cross-sectional study on a national level of patients admitted with index TEER procedures using the National Readmissions Database (NRD) (2016-2019). The NRD contains discharge summaries for nearly 18 million hospital stays per year. It is a part of the Healthcare Cost and Utilization Project, which the agency sponsors for healthcare research and quality.

Study criteria

The national US estimates were produced by using sampling weights provided by the NRD. We identified the index of TEER cases with anemia (hemoglobin [Hb] levels <12.0 g/dL in women and <13.0 g/dL in men) and without anemia using the International Classification of Disease, Tenth Edition, Clinical Modification (ICD-10-CM) [13].

Ethical considerations

We calculated readmission frequency with a national sample weighted at 30 days, 90 days, and 180 days post-TEER index procedure. Duplicates were removed. These data are deidentified and publicly available, thus exempt from institutional review board approval, and the need for informed consent was waived.

Procedure details

Transcatheter edge-to-edge repair is one of the novel percutaneous techniques used for the repair of mitral regurgitation. During the procedure, some portions of the anterior and posterior leaflets of the mitral valve are fused together. A 24F delivery sheath is advanced in the left atrium through the femoral vein. The device is advanced using the guiding catheter through the delivery sheath. 

Patient and hospital characteristics

Baseline patient characteristics, including demographics and clinically relevant comorbidities, are shown in Table 1. Our study included all patients admitted for TEER procedures with and without anemia, aged >18 years. Demographic information, including primary insurance payer, median household income, relevant comorbid conditions (i.e., diabetes, hypertension, hyperlipidemia, obesity, smoking, congestive heart failure, peripheral vascular disease, liver disease, and metastatic cancer), and hospital-level characteristics were collected.

**Table 1:** Baseline characteristics and comorbidities in patients with anemia vs without anemia who underwent TEER procedure. TEER: transcatheter edge-to-edge repair, CABG: coronary artery bypass graft, PCI: percutaneous coronary intervention.

Baseline characteristics	No anemia (n=27,561)	Anemia (n=1434)	p-value
Gender			
Male	15018 (54.5)	691 (48)	<0.05
Female	12545 (45.5)	744 (52)	
Year			
2016	4476 (16.2)	230 (16)	0.9604
2017	5781 (21)	314 (22)	
2018	7077 (26)	363.3 (25.3)	
2019	10229 (37)	527 (37)	
Rehab transfer			
No rehab transfer	27,414 (99.5)	1417 (99)	<0.05
Rehab transfer	149 (0.54)	18 (1.2)	
State resident status			
Non-resident	2690 (9.8)	107 (7.4)	0.0762
Resident	24,873 (90.2)	1328 (92.5)	
Same day event			
Not a transfer or same-day event	26,292 (95.4)	1234 (86)	<0.001
Transfer of 2 discharges from different hospital	714 (2.6)	123.5 (8.6)	
Same-day stay of 2 discharges from different hospital	258.3 (0.93)	48.6 (3.4)	
Same day stay of 2 discharges from same hospital	140.6 (0.5)	9.6 (0.66)	
Same-day stay of 3 discharges from same/different hospital	158 (0.6)	19 (1.33)	
Median household income $			
0–25th percentile	6004 (22)	332 (23.4)	0.3804
26–50th percentile	6972 (25.6)	392 (28)	
51–75th percentile	7446 (27.3)	359 (25.3)	
76–100th percentile	6834 (95.3)	335 (4.7)	
Hospital bed size			
Small	1137 (4.12)	39 (2.7)	0.2004
Medium	5687 (20.6)	268 (18.7)	
Large	20,739 (75.2)	1128 (78.6)	
Control/ownership of the hospital			
Government, non-federal	2256 (8.2)	127 (8.84)	0.4377
Private, non-profit	22,071 (80)	1121 (78)	
Private, invest own	3235 (11.7)	187 (13)	
Urban-rural hospital designation			
Large metropolitan	19,018 (69)	1064 (74.1)	<0.05
Small metropolitan	8408 (30.5)	368.4 (26)	
Micropolitan	134 (0.5)	2.7 (0.18)	
Not Metropolitan	2.9 (0.01)	0	
Teaching status of urban hospitals			
Metropolitan non-teaching	2611 (9.5)	128.4 (9)	0.436
Metropolitan teaching	24,814 (90)	1304 (91)	
Non-metropolitan	137 (0.5)	2.7 (0.2)	
Admission day			
Monday-Friday	26,503 (96)	1345 (94)	<0.001
Saturday-Sunday	1059 (4)	90 (6.25)	
Primary expected healthcare cost payer			
Medicare	24,083 (87.4)	1217 (85)	0.1162
Medicaid	661 (2.4)	43 (3)	
Private	2313 (8.4)	157 (11)	
Self-pay	119 (0.43)	7.6 (0.52)	
No charge	16 (0.05)	0	
Other	359 (1.3)	11 (0.74)	
Comorbidities			
Hyperlipidemia	16736 (61)	908 (63.3)	0.176
Hypertension	19,198 (70)	1126 (78.5)	<0.001
Smoker	9390 (34)	465 (32.4)	0.396
Prior myocardial infarction	4486 (16.3)	268 (19)	0.1014
Prior PCI	4881 (18)	279 (19.5)	0.2296
Prior CABG	5793 (21)	311 (22)	0.6807
OSA	3573 (13)	227 (16)	<0.05
Pulmonary disease	5916 (21.5)	374 (26)	<0.001
Pneumonia	584 (2.1)	50 (3.5)	<0.05
Hypothyroidism	4798 (17.4)	302 (21)	<0.05
Liver disease	451 (1.6)	38 (2.6)	<0.05
Obesity	3026 (11)	201 (14)	<0.05

Outcome measures

The primary outcome of our study was the readmission rate and in-hospital mortality due to TEER procedures with and without anemia. Secondary outcomes included heart failure (HF), acute kidney injury (AKI), stroke, myocardial infarction (MI), major adverse cardiac and cerebrovascular events (MACCE), and mechanical circulatory support (MCS) in anemic patients who had TEER procedures.

Statistical analysis

We used a confidence interval (CI) of 95% and a p-value <0.05 as statistically significant in all our analyses. Continuous variables were analyzed using means with standard deviations or medians with interquartile ranges for normally distributed and skewed data, respectively. The categorical variables were analyzed using descriptive statistics with frequencies and percentages. Patients' demographics, comorbidities, hospital characteristics, and in-hospital outcomes were compared between patients who underwent TEER procedures with and without anemia using the Pearson χ2 test for categorical variables and the independent sample t-test for continuous variables. The propensity score matching module (PSM-2) was applied to limit confounders. Univariate and multivariate logistic regression were used to calculate unadjusted and adjusted odds ratios for in-hospital clinical outcomes. The multivariate regression model was adjusted for baseline comorbidities and demographic variables to avoid confounding and effect modification. NRD hospital costs are adjusted with the national inflation index (www.bls.gov). All analyses were conducted using STATA v.17.1 TX (StataCorp, LLC, Texas, USA).

## Results

Demographics and baseline characteristics

A total of 28,995 patients who had undergone TEER in the United States between 2016 and 2019 were included in the final cohort. About 1,434 (4.9%) of these patients had anemia, whereas 27,561 (95.1%) had no diagnosis of anemia. The mean age of TEER-anemia and TEER without anemia was 76.9 ± 10.8 vs. 77.7 ± 10.2, respectively (Table 1). The majority of patients with anemia were female (n=744, 52%), while the majority of patients without anemia were male (n=15018, 54.5%). The most common comorbidity among both groups was hypertension (with anemia, N=1126 [78.5%]; without anemia, N=19198 [70%], p<0.0001, Table 1). A detailed description of the baseline demographics and comorbidities is provided in Table 1.

Univariate and multivariate regression outcomes among anemic and non-anemic patients

**Figure 1:**
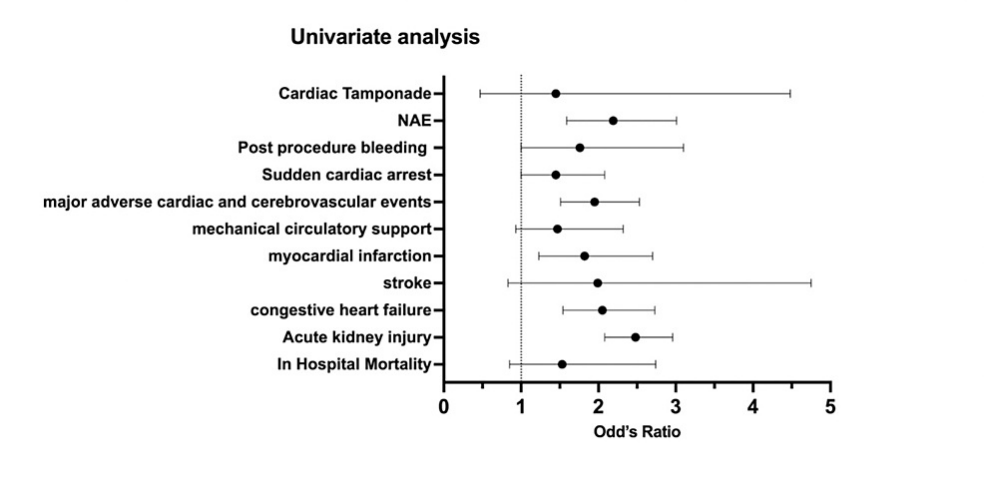
Univariate Cox regression analysis of the clinical outcome in patients underwent TEER procedure with anemia. TEER: transcatheter edge-to-edge repair.

In the unadjusted model, anemic patients had higher odds of AKI (OR 2.48; 95% [CI 2.08-2.96; p<0.001]), HF (OR 2.05; 95% [CI 1.54-2.73; p<0.001]), MI (OR 1.82; 95% [CI 1.23-2.7; p<0.001]), MACCE (OR 1.95; 95% [CI 1.51-2.53; p<0.001]), and net adverse events (OR 2.19; 95% [CI 1.59-3.01; p<0.001]). Other outcomes were not statistically significant (Table 2 and Figure 1).

**Table 2:** Univariate and multivariate regression analysis of the outcomes of TEER with anemia. TEER: transcatheter edge-to-edge repair, AKI: acute kidney injury, MI: myocardial infarction, MACCE: major adverse cardiac and cerebrovascular events, HF: heart failure, MCS: mechanical circulatory support.

	Univariate regression analysis			Multivariate regression analysis		
In-hospital outcome	OR	95% CI	p-value	aOR	95% CI	p-value
In-hospital mortality	1.53	0.85–2.74	0.15	1.39	0.76–2.53	0.272
AKI	2.48	2.08–2.96	<0.001	2.21	1.81–2.6	<0.001
HF	2.05	1.54–2.73	<0.001	1.75	1.28–2.3	<0.001
Stroke	1.99	0.83–4.75	0.12	1.85	0.75–4.54	0.177
MI	1.82	1.23–2.7	<0.001	1.54	1.01–2.33	0.041
MCS	1.47	0.93–2.32	0.096	1.3	0.81–2.08	0.272
MACCE	1.95	1.51–2.53	<0.001	1.72	1.29–2.3	<0.001
Post-procedure bleeding	1.76	1–3.1	0.047	1.72	0.97–3	0.059
NAE	2.19	1.59–3.01	<0.001	1.85	1.32–2.59	<0.001
Cardiac tamponade	1.45	0.47–4.48	0.517	1.27	0.38–4.21	0.689

When we adjusted for demographics and comorbidities, anemic patients had higher odds of AKI (aOR 2.21; 95% [CI 1.81-2.6; p<0.001]), HF (aOR 1.75; 95% [CI 1.28-2.3; p<0.001]), MI (aOR 1.54; 95% [CI 1.01-2.33; p<0.041]), MACCE (aOR 1.72; 95% [CI 1.29-2.3; p<0.001]), and net adverse event (aOR 1.85; 95% [CI 1.32-2.59; p<0.001]), and the association was statistically significant despite adjusting for confounders.

Propensity Match Score analysis

**Figure 2:**
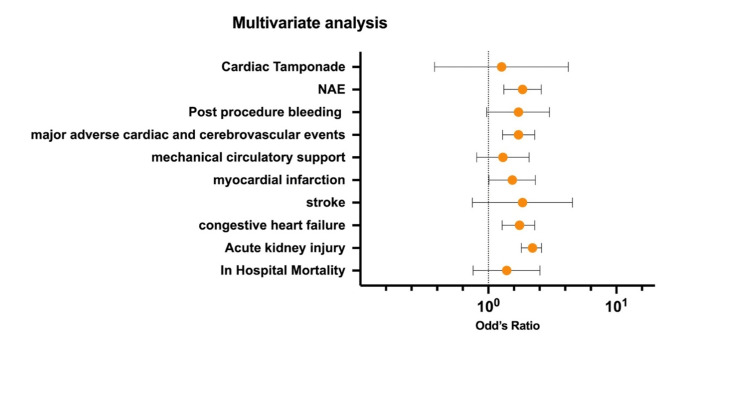
Multivariate Cox regression analysis of the clinical outcome in patients underwent TEER procedure with anemia.

After adjusting for baseline demographics and comorbidities, a matched sample was obtained on PSM-2 analysis (Table 3). Overall, the findings of the PSM analysis closely mirror the adjusted analysis, with some exceptions. TEER with the anemia group showed statistically significantly higher rates of AKI (30% vs. 20.4%), heart failure (91.6% vs. 87.3%), post-procedural bleeding (2.2% vs. 0.62%), and MACCE (12.8% vs. 8.5%). There was no statistically significant difference in the rate of in-hospital mortality (2.2% vs. 2%, p=0.257), stroke (0.74% vs. 0.12%, p=0.058), cardiogenic shock (8.4% vs. 7.1%, p=0.354), major circulatory support (3.5% vs. 3%, p=0.573), or cardiac tamponade (0.5% vs. 0.25%, p=0.413) (Table 3 and Figure 2). 

**Table 3:** Propensity matched analysis comparing TEER in anemic and nonanemic group. AKI: acute kidney injury, HF: heart failure, MI: myocardial infarction, MCS: major circulatory support, MACCE: major adverse cardiac and cerebrovascular events.

In-hospital outcome	No anemia (%)	Anemia (%)	P-value
In-hospital mortality	16 (2)	23 (2.8)	0.257
AKI	166 (20.4)	254 (30)	<0.01
HF	709 (87.3)	744 (91.6)	<0.05
Stroke	1 (0.12)	6 (0.74)	0.058
Cardiogenic shock	58 (7.1)	68 (8.4)	0.354
MI	19 (2.3)	29 (3.6)	0.143
Sudden cardiac death	22 (2.7)	43 (5.3)	<0.05
MCS	24 (3)	28 (3.5)	0.573
Post-procedural bleeding	5 (0.62)	18 (2.2)	<0.05
MACCE	69 (8.5)	104 (12.8)	<0.05
NAE	722 (89)	754 (93)	<0.05
Cardiac tamponade	2 (0.25)	4 (0.5)	0.413

Resource utilization

The length of stay (LOS) was longer in the anemic group than in the non-anemic group across all years. In addition, the readmission rate was higher overall in the anemic group at 30, 90, and 180 days from 2016 to 2019 (Tables 4-5).

**Table 4:** Readmissions at 30, 90, and 180 days of patients who underwent TEER with and without anemia.

Index admissions	No anemia (%)	Anemia (%)	Total (%)
2016			
Readmitted in 30 days	457.8 (15.3)	16 (8.3)	474 (15)
Readmitted in 90 days	374 (15.3)	15 (9.3)	388.4 (15)
Readmitted in 180 days	713 (15)	41 (12)	754 (15)
2017			
Readmitted in 30 days	695 (23)	45 (23)	739 (23)
Readmitted in 90 days	581 (24)	36 (22.5)	617 (24)
Readmitted in 180 days	1075 (23)	73 (21)	1148 (22.5)
2018			
Readmitted in 30 days	798.4 (27)	58 (30)	856.5 (27)
Readmitted in 90 days	636 (26)	48 (30)	684 (26.3)
Readmitted in 180 days	1211 (26)	97 (28)	1307 (26)
2019			
Readmitted in 30 days:	1046 (35)	76 (39)	1122 (35)
Readmitted in 90 days:	853.5 (35)	61 (38)	914.4 (35)
Readmitted in 180 days	1745 (37)	139 (40)	1,883 (37)

**Table 5:** Length of stay for 30, 90, and 180 days of readmission in patients who underwent MitraClip with and without anemia.

	Mean ± SD	
Time-window	No anemia	Anemia
30 days		
2016	4.2 ± 4.5	7 ± 7
2017	4.2 ± 4.4	3.7 ± 4
2018	4.3 ± 5.3	7.3 ± 5.8
2019	4.3 ± 5	6.8 ± 5.8
90 days		
2016	6 ± 8.1	8.2 ± 8
2017	5.8 ± 7.8	8 ± 7.4
2018	5.1 ± 7.2	9.1 ± 8.7
2019	5.7 ± 8.7	8 ± 7
180 days		
2016	6.3 ± 9.3	7.1 ± 7
2017	5.9 ± 9.4	9.7 ± 8.2
2018	5 ± 7.6	10 ± 13
2019	5.5 ± 9.2	9.1 ± 8.5

## Discussion

We have assessed the clinical outcomes and readmission rate in patients with anemia who had undergone TEER placement due to severe MR. Our results showed that patients with anemia had a longer hospital stay post-TEER procedure compared to the non-anemic cohort. They also had a higher incidence of AKI, heart failure, sudden cardiac death, post-procedural bleeding, and MACCE.

Transcatheter mitral valve repair with TEER has been shown to be safe in elderly patients with multiple comorbidities who cannot undergo mitral valve surgery due to prohibitive surgical risk [14]. However, data on the influence of preprocedural anemia on clinical outcomes in patients undergoing TEER procedures is limited at present. Anemia has been found to be associated with adverse clinical outcomes in patients undergoing percutaneous coronary interventions, cardiac surgery, and transcatheter aortic valve implantation (TAVI) [8,15,16]. Anemia is common in the elderly, with a prevalence ranging from 8% to 44%. Anemia from chronic disease and iron deficiency are the most common causes of anemia in the geriatric population [17]. A prospective observational study by Iliadis et al. found the prevalence of iron deficiency to be around 52% in patients undergoing TEER [18]. Among the patients in the anemic subgroup in our analysis, 93.5% had anemia related to iron or vitamin deficiencies, highlighting a critical modifiable risk factor for better outcomes post-procedure.

A meta-analysis of 230,795 patients who had undergone PCI revealed an elevated risk of MACE in patients with anemia compared to those who did not (RR 2.39, CI 2.02-2.83, p<0.001) [19]. Our study did not show a difference in mortality; however, in an analysis of 749 patients enrolled in the transcatheter mitral valve interventions (TRAMI) registry, anemia was found to be a strong predictor of 1-year mortality (HR 2.44, p=0.02) [20]. Another prospective observational study of 130 patients undergoing a mitraclip procedure found anemia associated with a higher risk for a combined end-point of mortality and heart failure hospitalizations (HR: 2.51; 95% CI: 1.24-5.09; P = 0.01) [15]. Anemic patients in our study had higher odds of heart failure, consistent with these studies, even after a multivariate regression analysis. 

Patients in the anemia subgroup in our analysis had a higher incidence of AKI and MI post-TEER placement. The incidence of AKI after TEER is high and occurs in around 20% of patients undergoing this procedure, despite being a zero-contrast procedure [21]. A recent observational study of 721 patients who had undergone TEER implantation found anemia (defined as Hb<11 g/dl) to be an independent predictor of AKI (OR 1.97, p=0.003) [22]. Studies have shown the prevalence of anemia in patients with MI ranges between 10% and 27% and has poor clinical outcomes [23-26]. Anemia is also a common comorbidity in patients with stroke, with an estimated prevalence of 26.9% (95% CI: 13.6-30.3%) in a recent meta-analysis of 19,239 patients treated for a stroke. Anemia was found to be independently associated with an increased risk of mortality in this patient population (adjusted OR = 1.39, 95% CI: 1.22-1.58) [27].

Due to the paucity of studies on the impact of pre-procedural anemia in patients undergoing TEER placement, data from similar studies done in patients undergoing TAVI help interpret our findings. Both groups consist of older patients with multiple comorbidities and a high surgical risk, preventing them from undergoing valve replacement surgeries.

Limitations

Due to a lack of follow-up data, our study is unable to evaluate the impact of anemia after the discharge of patients. Since our data were derived from a retrospective registry, it is likely that the data suffers from selection bias. Additionally, the device type that was used for the TEER procedure was not documented in the database. Furthermore, the NRD lacked information such as racial demographic information, lab values, and echocardiographic parameters (e.g., ejection fraction, left atrial volume index, etc.).

Despite the possible inclusion of coding errors and disparities in documentation, the NRD database has been broadly used and validated. A nationally represented database was used to extract a large sample size, which included data from multiple hospitals and populations across 23 states in the USA. Finally, the severity of anemia will likely result in worse clinical outcomes in patients undergoing TEER. However, our analysis did not assess such an association; further detailed analysis with a larger number of populations is needed to establish such a hypothesis.

## Conclusions

The diagnosis and management of anemia play a pivotal role in the clinical care of patients with severe MR who are undergoing TEER. Our investigation revealed a significant correlation between anemia and adverse clinical outcomes in this patient cohort. Furthermore, individuals diagnosed with anemia who underwent TEER procedures experienced notably prolonged hospital stays when compared to their non-anemic counterparts. This association underscores the importance of early identification and effective management of anemia as part of the preoperative evaluation of patients slated for TEER to potentially mitigate risks and improve postoperative recovery trajectories.
